# Punicalagin Regulates Signaling Pathways in Inflammation-Associated Chronic Diseases

**DOI:** 10.3390/antiox11010029

**Published:** 2021-12-24

**Authors:** Jie Xu, Ke Cao, Xuyun Liu, Lin Zhao, Zhihui Feng, Jiankang Liu

**Affiliations:** 1 Center for Mitochondrial Biology and Medicine, The Key Laboratory of Biomedical Information Engineering of Ministry of Education, School of Life Science and Technology, Xi’an Jiaotong University, Xi’an 710049, China; ff.89.03.22@stu.xjtu.edu.cn (J.X.); caoke@stu.xjtu.edu.cn (K.C.); xuyunliu@mail.xjtu.edu.cn (X.L.); zhaolin2015@mail.xjtu.edu.cn (L.Z.); 2 Center for Mitochondrial Biology and Medicine, Frontier Institute of Science and Technology, Xi’an Jiaotong University, Xi’an 710049, China; zhfeng@mail.xjtu.edu.cn; 3 University of Health and Rehabilitation Sciences, Qingdao 266071, China

**Keywords:** pomegranate, polyphenols, punicalagin, inflammation-associated disease, signaling pathway

## Abstract

Inflammation is a complex biological defense system associated with a series of chronic diseases such as cancer, arthritis, diabetes, cardiovascular and neurodegenerative diseases. The extracts of pomegranate fruit and peel have been reported to possess health-beneficial properties in inflammation-associated chronic diseases. Punicalagin is considered to be the major active component of pomegranate extracts. In this review we have focused on recent studies into the therapeutic effects of punicalagin on inflammation-associated chronic diseases and the regulatory roles in NF-κB, MAPK, IL-6/JAK/STAT3 and PI3K/Akt/mTOR signaling pathways. We have concluded that punicalagin may be a promising therapeutic compound in preventing and treating inflammation-associated chronic diseases, although further clinical studies are required.

## 1. Introduction

Inflammation is a response to stimuli, either internal or external. The first protective response of a body’s immune system to inflammation can be divided into the acute phase and the chronic phase [1]. The acute phase is characterized by increased blood flow and vascular permeability, and accumulation of leukocytes and cytokines, while the chronic phase is characterized by the development of specific humoral and cellular immune responses [1]. If acute inflammation is failed to regulate, it will lead to chronic inflammation [2]. The system of inflammation pathway consists of inducers, sensors, mediators and effectors [3]. According to the different stimuli, inflammation can be classified as pathogen-associated molecular patterns and damage-associated molecular patterns. These inducers can be recognized by different pattern recognition receptors in macrophages and dendritic cells. Then, inflammatory cytokines are released and immune cells are recruited. The immune cells will release enzymes to fight off the infectious objects and clear death cells [4]. Any imperfection of an inflammatory response may cause disease [2]. Excess inflammation responses lead to diseases such as osteoarthritis [5], rheumatoid arthritis [6], or gastric ulcers [7]. As a consequence of inflammation, reactive oxygen species (ROS) will accumulate and damage healthy cells [3]. Chronic inflammation is also a feature that is common to atherosclerosis, Parkinson’s disease [8], Alzheimer’s disease [9] and diabetes [10].

**Figure 1:**
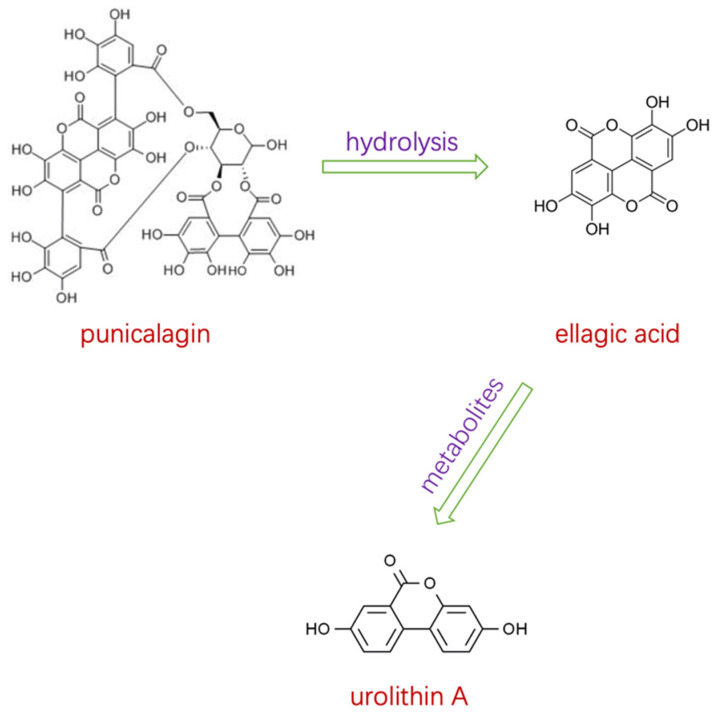
Punicalagin is hydrolyzed into ellagic acid in the small intestine, and is then metabolized to urolithin A.

Natural compounds from plants have garnered increasing attention among the scientific community for their lower cost, higher bioavailability, and less toxicity compared to synthetic pharmaceutical agents [11]. Polyphenols are widely found in vegetables and fruits in nature [12]. As an important source of anthocyanins and hydrolysable tannins, pomegranate is consumed as a fruit and is also used for its antioxidant and anti-inflammation potential on disease prevention and treatment [13]. Pomegranate peel extract contains high amounts of bioactive compounds, mainly phenolic acids, flavonoids and tannins [14]. Among all the polyphenols in pomegranate, punicalagin, [2,3-(S) hexahydroxydiphenoyl-4,6-(S,S)-gallagyl-d-glucose] (structure shown in Figure 1), is the richest and most active one. Punicalagin has been reported to have beneficial effects on both chronic inflammation [15] and acute inflammation [16], and to be involved in different steps in inflammation, including immune response [17], cells macrophages [18,19] and fibroblasts [20], and necrosis [17]. For example, punicalagin downregulated the mRNA and soluble protein expression of IL-2 from anti-CD3/anti-CD28 stimulated murine splenic CD4+ T cells, and inhibits the activation of the nuclear factor of activated T cells [17].

Considering the effective protective effects of punicalagin on various diseases, we aim to have a comprehensive review of the regulatory roles of signaling pathways of punicalagin on inflammation-associated chronic diseases.

## 2. Punicalagin Metabolism and Epidemiology

Pomegranate (*Punica granatum* L.) is a plant native to Asia and is now widely distributed in subtropical and tropical regions around the world [21]. Pomegranate has been well-known for its medical use for centuries. The history of cultivation and consumption of pomegranate can be dated back to 3000 BC [22]. It is documented in traditional Chinese medicine and other traditional medicines, including Indian, Cuban and Greek traditional medicine [23]. It is consolidated that the oxidative stress is present in all life levels, with different regulatory mechanisms, from bacteria to human health. Since pomegranate is rich in antioxidants, pomegranate has been proposed to have potential beneficial effects on human health [24]. The nutritional and health properties of pomegranate are not limited to its edible parts, but also include non-edible parts [25]. In fact, pomegranate fruits and inedible parts of trees (peel, flowers, etc.) contain higher levels of active ingredients [26].

The health properties of pomegranate has led to the expansion of the pomegranate industry worldwide. The global total production is nearly 4.5 million tons. The pomegranate peel accounts for about 40% of the total weight of the pomegranate fruit [27]. As a by-product of the pomegranate industry, pomegranate peel has been considered an agro-industry waste for a long time [28], until researchers found that the bioactive compounds in the peel is higher than in the aril and seeds [29]. Nearly fifty phenolic compounds have been found in pomegranate peel, including flavonoids such as anthocyanins, catechins, and hydrolyzable tannins such as punicalagin, gallic acid and ellagic acid (Table 1) [30]. Various methods have been described for the extraction of polyphenols from pomegranate [31,32,33,34]. Different solvents on extraction from pomegranate influence the phenolic content and antioxidant properties. Compared with non-polar solvents, polar solvents have a stronger antioxidant extraction ability. Methanolic pomegranate peel extracts have been proven to be superior over other solvent extracts [29]. However, after consideration of safety concerns, ethanol was preferred over methanol [35].

Empirical studies have shown that the hydrolyzed polyphenols in pomegranate peel possess very important nutritional and medicinal values for its numerous biological activities, especially high levels of antioxidant activity [36]. Punicalagin is the most abundant bioactive compound, with a high molecular weight isolated from pomegranate peel (Table 1). The major antioxidant activity of pomegranate juice is from the polyphenol ingredients, especially punicalagin [37]. The content of punicalagin in pomegranate peel is the highest among common fruits. Studies have shown that the content of punicalagin in pomegranate peels is 10–50 mg/g [29]. Moreover, the concentration processes did not affect the punicalagin content, which showed that pomegranate juices from concentrate can also provide health benefits [38].

Punicalagin is soluble in water and is the precursor of ellagitannin, accounting for 85% of the tannins in pomegranate peel (*w*/*w*) [39]. As a hydrolyzable tannin, punicalagin can be hydrolyzed spontaneously into ellagic acid in vivo. Then, ellagic acid can be transformed by gut microbiota to urolithins A [40] (Figure 1). Although the promising therapeutic effects of punicalagin have been shown in lots of in vitro studies, the bioavailability testing of pomegranate ellagitannins still requires further study [41].

Punicalagin has a variety of biological effects, including antioxidant [42,43], antiviral [44] and antimicrobial [45,46,47] activities (Table 2). Studies have shown that punicalagin could also significantly inhibit oxidative DNA damage. Punicalagin has even been reported to exert a protective effect against high glucose-induced neural tube defects [48]. At the same time, polyphenols are also important anti-cancer agents because of the ability of anti-mutation and anti-proliferation. Therefore, several studies have linked punicalagin with anti-cancer activity [20,49]. In vitro, it has been found that punicalagin could inhibit more than 90% of the mutagenesis caused by benzo [a] pyrene in female SD rat lung [50]. Other studies have also found that punicalagin could inhibit the proliferation of prostate cancer cells by inducing apoptosis and anti-angiogenesis [51]. Our recent study investigated the effect of punicalagin on endothelial dysfunction and showed that punicalagin enhanced FoxO1 nuclear translocation and that silencing FoxO1 remarkably abolished the ability of punicalagin to augment the mitochondrial biogenesis, eNOS expression and oxidative stress, leading to amelioration of endothelial dysfunction [52]. We have also reported the potent protective effects of punicalagin on acute hyperlipidemia-induced hepatic lipid metabolic disorders [53], and neurotoxicity and AMPK activation in hippocampal neurons [54]. Recently, a study evaluated the ability of pomegranate peel extract polyphenols as anti-SARS-CoV-2 agents and showed that punicalagin exhibited a higher affinity than the positive controls umifenovir and lopinavir for the predicted druggable active site on the SARS-CoV-2 protein target [55].

Punicalagin has been considered the main active component among the polyphenols in pomegranate peel extract in anti-inflammation [56]. Since inflammation is the cause of many disease [57,58,59], the potential anti-inflammatory activity may be important to explain the health-promoting activity of punicalagin.

## 3. Role of Punicalagin in Inflammation-Associated Diseases

Inflammation is a complex and necessary component of the defense system of an organism against biological, chemical, and physical stimuli [60]. In the 19th century, the link between inflammation and the development of cancer has been found [61,62]. Hereafter, abundance of evidence emphasizes the importance of inflammation in the development of chronic disease [63]. It is generally described as consisting of acute and chronic phases. The acute inflammation is involved in infectious disease [64]. Persistent inflammation can lead to the chronic phase [65]. Chronic inflammation contributes to immune diseases [66], arthritis, diabetes, cancer [67], cardiovascular and neurodegenerative diseases [8,68] and many other chronic diseases [69,70,71].

The inflammatory process involves lots of signaling pathways and cytokines. The first step of inflammation is to specifically recognize the pathogens which are mediated by the pathogen-associated molecular patterns and damage-associated molecular patterns [72]; the second step is to activate specific immune signaling pathways to promote the secretion of pro-inflammatory cytokines, such as interleukin-1-beta (IL-1β), IL-6, tumor necrosis factor-alpha (TNF-α) [60]. These events recruit immune cells resulting in the generation of reactive oxygen species (ROS) to activate a series of signaling pathways. In this review, we will focus on the following classical inflammatory pathways: NF-κB, mitogen-activated protein kinase (MAPK), IL-6/JAK/STAT3 and phosphatidylinositol-3kinases/Akt/mammalian target of the rapamycin (PI3K/Akt/mTOR) signaling pathways.

### 3.1. Effect on IL-6/JAK/STAT3

Cytokines participate in many fundamental processes of life, including immune response, inflammation, metabolism, etc. IL-6, a pleiotropic cytokine, plays an important role in inflammation [73]. Levels of IL-6 are increased in chronic inflammatory conditions. Janus kinases and tyrosine kinase 2, along with activators of transcription (STAT) signaling, are major factors in pro- and anti-inflammatory cytokine signaling [74]. The high level of IL-6 stimulates the activation of the JAK/STAT3 pathway [75,76].

In a recent study, Cao, et al. [77] used LPS exposure to activate RAW264.7 cell inflammation reflection. After 24 h LPS stimulation, IL-6 and TNF-α secretion in the supernatants was significantly enhanced. Pre-treatment with punicalagin (50 µM) and then treatment with LPS, significantly inhibited the secretion of IL-6 and TNF-α, indicating that punicalagin exerted anti-inflammatory activity via the suppression of NO production and pro-inflammatory cytokines IL-6 and TNF-α in LPS-induced RAW264.7 cells. It is worth noting that treatment with punicalagin only, without LPS treatment, had no effect on the basal level of IL-6, and TNF-α secretion in RAW 264.7 cells.

Ankylosing spondylitis is a chronic, progressive inflammatory disease. The exact mechanism of the ankylosing spondylitis pathogenesis is still under investigation, but lots of studies have reported that ankylosing spondylitis seemed to involve a variety of factors. The activated JAK/STAT3 signaling pathway and increased levels of ROS both were found to be involved in pathological formation of ankylosing spondylitis [78,79]. In ankylosing the spondylitis mouse model, ROS and malonaldehyde levels were increased, and punicalagin treatment significantly reduced ROS and malonaldehyde levels, and effectively improved antioxidant status in ankylosing spondylitis BALB/c mice [80]. This effect may be conducted by regulating the major pathway of inflammatory response JAK/STAT3 signaling [80]. In other research, punicalagin (250 mg/kg) pretreated with concanavalin A-induced autoimmune hepatitis mice down-regulated the levels of IL-6, TNF-α and IFN-γ, and reduced the infiltration of activated CD4^+^ and CD8^+^ T cells in liver [81]. Punicalagin (2.5 µg/mL) supplement down-regulated levels of IL-6, TNF-α and IL-1, suggesting that punicalagin could attenuate the inflammation caused by influenza A virus in Madin-Darby Canine Kidney cells [82].

### 3.2. Effect on NF-κB Pathway

NF-κB has been considered a typical pro-inflammatory pathway for a long time. It has been defined in response to TNF-α and IL-1 signaling [83]. In the absence of an activating stimulus, IκBs binds and sequesters NF-κB dimers in the cytoplasm, masking their nuclear localization signal. Once the activating signal is received, the IκB proteins have rapid polyubiquitylation and degradation, liberating NF-κB dimers to translocate into the nucleus and regulate gene expression [84].

The adverse effect of chemotherapeutic drugs limits their clinical applications. Cisplatin is an agent that is used for the treatment of lung cancer, ovarian cancer and many other cancers [85]. It could induce acute kidney injury by elevating ROS [86] and activating different signaling pathways, such as NF-κB or IL-6 [87]. Punicalagin attenuated tissue injury by downregulating pro-inflammatory mediators NF-κB, TNF-α, IL-6 and enhancing antioxidant defenses via up-regulating Nrf2 [88]. Meanwhile, in the human osteoblast cell line (hFOB1.19) and three human osteosarcoma cell lines (U2OS, MG63 and SaOS2), punicalagin degraded IκBα and the nuclear translocation of p65, suggesting an attenuation of the NF-κB signaling pathway [89]. In other research, Zhang et al. [90] found that punicalagin suppressed NF-κB activity in the cervical cancer cell ME-180. Punicalagin has also been reported to possess an anti-cancer activity of papillary thyroid carcinoma, the most common endocrine carcinoma [91]. In other research concerning papillary thyroid carcinoma, punicalagin exposure caused the phosphorylation and subsequent degradation of IκBα and the nuclear translocation of p65, indicating the regulating role of punicalagin in the NF-κB signaling pathway [92]. Mukherjee et al. [93] reported that pomegranate polyphenols including punicalagin and ellagic acid supplementation in bearing mice modulated Nrf2 and NF-κB and decreased tumor-induced hepatic damage and cell death.

### 3.3. Effect on MAPK Pathway

The MAPK signaling pathway consists of a series of cross-talking and compensatory pathways in cellular metabolism [94]. There are three main classical MAPKs: ERKs, JNKs, and p38 MAPKs [95]. In a study dedicated to exploring the anti-inflammatory mechanism of polyphenols in pomegranate peel, researchers found that in RAW264.7 marcrophages, punicalagin significantly decreased the production and gene expression of pro-inflammatory cytokines triggered by LPS. The inhibitory effects were attributable to suppression of p38, ERK and JNK phosphorylation levels in MAPK signaling pathway [56].

Systemic lupus erythematosus is a common autoimmune disease. Lupus nephritis is the most serious complication of systemic lupus erythematosus. The pathogenic of lupus nephritis is closely related to protease-activated receptor-2 (PAR2) [96]. PAR2 could enhance the production of inflammatory cytokines by activating ERK/MAPK pathways [97]. Recently published research reported that punicalagin had beneficial effects on lupus nephritis and this effect may be through the potent inhibition of PAR2-mediated activation of the ERK1/2 signaling pathway [98]. The receptor activator of NF-κB ligand (RANKL) is the key molecule required for osteoclast differentiation [99]. In a project, the researchers investigated the effect of punicalagin on osteoporosis and found that punicalagin treatment inhibited RANKL-induced osteoclast formation in vitro and attenuated ovariectomized-induced bone destruction in vivo [100]. Punicalagin treatment decreased the levels of p-JNK, indicating that punicalagin interfered with the MAPK pathway activation [100]. The anti-inflammation potential of punicalagin was also exhibited on cattle. Research into bovine endometritis using lipopolysaccharide (LPS) induced bovine endometrial epithelial cells to investigate the effect of punicalagin. The result showed that punicalagin pretreatment significantly decreased the productions of IL-1β, IL-6 and IL-8. Molecular mechanistic studies showed that punicalagin suppressed the phosphorylations of p38, c-JNK and ERK, suggesting that punicalagin could inhibit LPS-induced MAPK activation [101].

### 3.4. Effect on PI3K/AKT/mTOR Pathway

The mTOR pathway is indispensable for many cellular biological processes. In recent years, the PI3K/Akt/mTOR signaling pathway has emerged as a critical pathway in regulating the inflammatory response [102]. In research whose purpose was to investigate the role of mTOR in pomegranate-mediated anti-inflammation, Sprague-Dawley rats received 57 mL/day pomegranate juice rich in punicalagin. The results showed that pomegranate juice significantly downregulated pro-inflammatory enzymes nitric oxide synthase and cyclooxygenase-2 mRNA and protein expression. In addition, it inhibited phosphorylation of PI3K/AKT and mTOR expression, suggesting that punicalagin may affect the mTOR pathway [103]. This pathway is often dysregulated in cancer patients. It is one of the most important signaling pathways in cancer progression including proliferation, apoptosis, angiogenesis, and drug resistance [104,105]. Several studies have identified the beneficial effect of punicalagin on different cancers. Cheng et al. [91] reported that punicalagin treatment decreased the viability of thyroid cancer cell line BCPAP by activating the MAPK and inhibiting the mTOR signaling pathways to promote the process of autophagy. Recent research [106] reported a comparison between the effect of pomegranate peel extracts and pomegranate juice in prostate cancer DU-145 and PC-3 lines. The main phenolic compounds identified in the pomegranate peel extract of this project is α, β-punicalagin and ellagic acid. The results showed that the extracts of pomegranate peel had an important anti-cancer effect against prostate cancer cells by modulating the mTOR/S6K signaling pathway. As a metabolite of punicalagin [107], ellagic acid has been reported to inhibit tumor proliferation. In research concerning cervical cancer, 2.5 μM ellagic acid treatment inhibited the AKT/mTOR signaling pathway by enhancing the expression level of IGFBP7, which could inhibit the invasion of HeLa cells [108]. As shown in Figure 1, punicalagin can be hydrolysis into ellagic acid, then ellagic acid is metabolized to urolithin A and B by the intestinal microbiota in vivo [109]. Totiger et al. [110] found that treatment of pancreatic ductal adenocarcinoma cells with urolithin A blocked the phosphorylation of AKT and p70S6K in vitro, and successfully inhibited the growth of tumor xenografts, and increased the overall survival of *Ptf1a^Cre^*^/*+*^; *LSL-Kras^G12D^*^/*+*^; *Tgfbr2^flox^*^/*flox*^ (PKT) mice.

The mTOR signaling pathway has also been the focus of aging research [111]. Accumulated evidence has indicated that mTOR signaling pathways play an important role in cellular aging [112]. Although there is no direct report on punicalagin, the ability of pomegranate extract to improve aging-related diseases by regulating the mTOR pathway has been extensively studied. Alzheimer’s disease is the primary cause. In 2016, Bradidy et al. [113] fed mice with a 4% pomegranate diet for 15 months and found that the treatment reduced the expression of inflammatory genes and increased the phosphorylation levels of Akt and p70 S6 kinase in the APPsw/Tg mouse brain, suggesting that a pomegranate supplement could reduce neuroinflammation by activating the PI3K/Akt/mTOR signaling pathway.

## 4. Conclusions and Prospects

**Figure 2:**
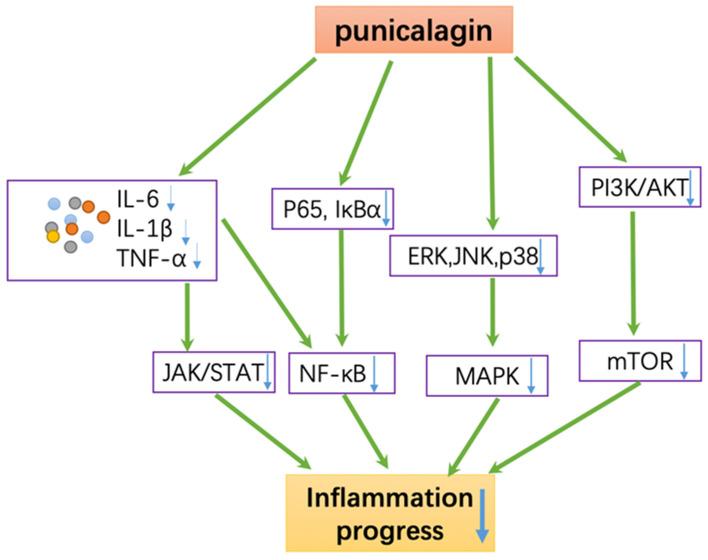
Various molecular targets of punicalagin in inflammation.

Inflammation is the development of chronic pathologies such as cancer, arthritis, diabetes, canrdiovascular and neurodegenerative diseases. Therefore, a drug or a therapy which has the ability to regulate inflammation means it has the possibility to improve chronic diseases. However, most of the current therapies cannot solve the problem fundamentally; therefore, there is an urgent need for searching better therapies. Preventive effects of punicalagin and its metabolites are mediated by several signaling pathways against inflammation including IL-6/JAK/STAT3 PI3K/Akt/mTOR, NF-κB, MAPK, and many other pathways (Figure 2).

These mechanisms provide strong evidence to support the fact that punicalagin may be able to comprehensively improve the inflammation-associated chronic diseases; however, the following issues should be considered seriously in future studies: (1) The information on the gastrointestinal fate of punicalagin and the cellular uptake of the bioactive compounds are still unclear and need to be explored; (2) Researchers mainly measure the effects and propose the pathway, but studies on the real molecular interactions are urgently required; and (3) Although a lot of basic studies have been carried out in laboratories, more clinical studies are needed to develop therapeutic strategies of punicalagin.

## Figures and Tables

**Table 1:** The major phenolic compounds in pomegranate peel.

Compound Name	Molecular Formula	Content (mg/g)
Punicalagin	C_48_H_28_O_30_	10–50
Ellagic acid	C_14_H_6_O_8_	1.2–5.8
Punicalin	C_34_H_22_O_22_	2–8
Catechin	C_15_H_14_O_6_	0.2–0.9
Chlorogenic acid	C_16_H_18_O_9_	0.4–3
Gallic acid	C_7_H_6_O_5_	0.2–4
Epicatechin	C_15_H_14_O_6_	0.9–2
Caffeic acid	C_9_H_8_O_4_	0.3–0.7
Ferulic acid	C_10_H_10_O_4_	0.46
Vanillic acid	C_14_H_18_O_9_	0.07
Rutin	C_27_H_30_O_16_	0.0045

**Table 2:** The biologic effect of punicalagin.

Activity of Punicalagin	Model	Experimental Outcome	Ref.
Antioxidant	l-NAME induced hypertension pregnant rats	Punicalagin supplement decreased the levels of oxidative stress	[42]
	CCl_4_-induced mice liver injury	Punicalagin decreased MDA level, increased SOD, GPX activities and Nrf2 expression	[43]
Anti-viral	Epithelial Vero host cell	Punicalagin reduction the virucidal plaque of HSV-1	[44]
Anti-microbial	*Aspergillus flavus* CECT2686, *Aspergillus parasiticus* CECT 2947, etc.	Pomegranate peel methanolic extracts inhibited the growth of *Aspergillus flavus*, *Fusarium verticillioides*, *Alternaria alternata* and *Botrytis cinerea*.	[45]
	*Staphylococcus aureus*	Punicalagin increased potassium efflux and exerted inhibitory effect on biofilm formation of *Staphylococcus aureus.*	[47]
Anti-cancer	Colorectal cancer cell HCT116	Punicalagin exhibits selective cytotoxicity on HCT116 compared to CCD841, exerts anti-cancer effect by downregulated Anx-A1 protein.	[49]

l-NAME: NG-nitro-L-arginine methyl ester; CCl-4: Carbon tetrachloride; HSV: Herpes simplex virus.
